# The CD20-specific engineered toxin antibody MT-3724 exhibits lethal effects against mantle cell lymphoma

**DOI:** 10.1038/s41408-018-0066-7

**Published:** 2018-03-20

**Authors:** Shengjian Huang, Changying Jiang, Hui Zhang, Taylor Bell, Hui Guo, Yang Liu, Yixin Yao, Dongfeng Zeng, Makhdum Ahmed, Krystle Nomie, Leo Zhang, Michael Wang

**Affiliations:** 1 Departments of Lymphoma and Myeloma, The University of Texas MD Anderson Cancer Center, Houston, TX USA; 2 Stem Cell Transplantation and Cellular Therapy, The University of Texas MD Anderson Cancer Center, Houston, TX USA

Mantle cell lymphoma (MCL) is a non-Hodgkin lymphoma (NHL) subtype with aggressive clinical demonstration characterized by the expression of neoplastic B-cell markers such as CD5, CD19, CD20, CD79, and BSAP/PAX5^1,2^. Notably, CD20 is strongly expressed by neoplastic B cells, enabling this cell surface protein to be exploited as a therapeutic target^3^. Targeting CD20 with anti-CD20 monoclonal antibodies such as rituximab has shown clinically meaningful outcomes^4–7^. To enhance antibody-mediated therapy, immunotoxins are utilized as an innovative cancer therapy tool that links an antibody or antibody fragment with a toxin, selectively localizing the toxin to the target cells to induce lethality^8^.

MT-3724, an engineered toxin body (ETB) comprised of a modified cytotoxic Shiga-like toxin 1A (3F7) and a CD20-specific single-chain variable fragment (scFv), recognizes CD20-expressing cells and triggers protein synthesis inhibition and apoptosis^9–12^. Although targeted therapy such as Bruton’s tyrosine kinase (BTK) inhibition by ibrutinib has achieved high response rates (68%) in relapsed/refractory MCL, therapeutic resistance has emerged as a barrier to improved patient outcomes and survival^13^. MT-3724 has the potential to bypass possible resistance mechanisms mediated via acquired BTK mutations or the activation of alternative survival signaling pathways by inhibiting tumor growth and survival through toxin-mediated activity^14,15^. To assess the anti-MCL effects of MT-3724, we tested its in vitro and in vivo efficacy in MCL cell lines and patient-derived xenograft (PDX) mouse models.

To correlate MT-3724 cytotoxicity with CD20 expression, CD20 surface expression was examined across 8 MCL cell lines (Supplementary Fig. S1A), and the CD20 MFI varied among different cell lines (Supplementary Fig. S1B and Supplementary Table S1). Four cell lines were treated with two MT-3724 doses for 24 h, resulting in undetectable CD20 expression, suggesting complete occupation of CD20 with MT-3724 (Supplementary Fig. S1C). We next verified whether MT-3724 induces cytotoxic activity against MCL. Indeed, MT-3724 inhibited the growth of MCL cell lines dose dependently (Fig. 1a), with the MT-3724 IC_50_ value ranging from 78 to 1383 ng/mL (Supplementary Table S1). No negative correlation between the IC_50_ and CD20 MFI was observed among the MCL cell lines (Supplementary Fig. S1D). However, no significant difference in the MT-3724 IC_50_ values was observed among the ibrutinib-sensitive and ibrutinib-resistant cell lines (Fig. 1b). Furthermore, 300 ng/mL MT-3724 was sufficient to reduce cell growth over time (Fig. 1c).

**Fig. 1:**
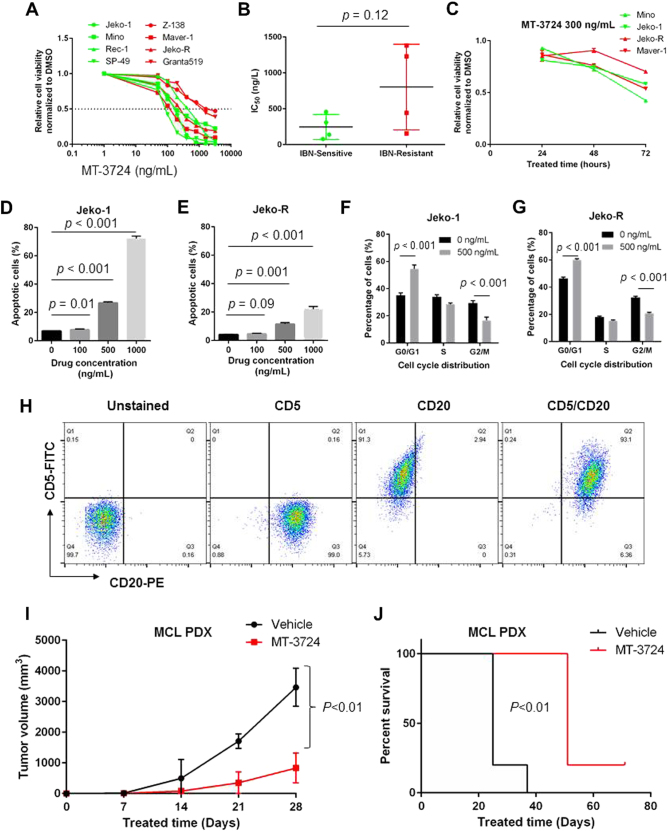
MT-3724 inhibits the growth of MCL cells in vitro and in vivo. **a** Cell viability of 8 MCL cell lines following 72 h treatment with the indicated increasing concentrations of MT-3724 (ibrutinib-sensitive cell lines: green; -resistant cell lines: red). **b** Comparison of the MT-3724 IC_50_ values among ibrutinib-sensitive (green) and –resistant (red) cell lines. **c** Time-dependent cell viability analysis (24 h, 48 h and 72 h assays) of 4 MCL cell lines treated with the indicated concentrations of MT-3724 (ibrutinib-sensitive cell lines: green; ibrutinib-resistant cell lines: red). **d, e** Apoptosis induction in Jeko-1 and Jeko-R cells treated with the indicated doses of MT-3724 for 24 h as measured by Annexin V/PI staining and flow cytometry. **f, g** Cell cycle arrest measured by PI staining in cell lines treated with 500 ng/mL MT-3724. Each treatment for cell viability, apoptosis and cell cycle was set up triplicate and conducted at least 3 independent times. **h** Immunophenotyping of MCL PDX tissue was conducted by two-color flow cytometry. Cells derived from the PDX were labeled CD5, CD20 single antibody or antibody combination. **i** Efficacy of single agent MT-3724 in a MCL PDX mouse model. PDX mice were treated IP with 1.2 mg/kg/dose MT-3724 or vehicle control for 5 days/week every other week for 4 weeks. Tumor volume was measured every week. *N* = 5 per treatment group. **j** Kaplan-Meier survival curves of the individual groups of mice. P values less than 0.05 indicate statistical significance. IBN: ibrutinib

Shiga toxin triggers mitochondrial stress *via* various cellular mechanisms such as decreasing anti-apoptotic protein levels, including MCL-1 and BCL-2^16–18^. To investigate whether MT-3724 induces apoptosis or cell cycle arrest in MCL, one pair of cell lines (Jeko-1 and Jeko-R) was treated with different MT-3724 doses for 24 h. As previously reported, Jeko-R is an acquired ibrutinib-resistant MCL cell line generated through chronic exposure to low ibrutinib concentrations^19^. MT-3724 induced apoptosis, and the percentage of apoptotic cells (Fig. 1d–e) and caspase 3/7 expression (Supplementary Fig. S2A-B) correlated with dosage in both cell lines. MT-3724-induced PARP cleavage and reduced BCL-2 and MCL-1 expression dose dependently (Supplementary Fig. S2C). MT-3724 has been suggested to inhibit protein synthesis and block the cell cycle^20^; therefore, we assessed the cell cycle effects of MT-3724 and found that both Jeko-1 and Jeko-R cells arrested in G0/G1 (Fig. 1f, g). To evaluate the in vivo efficacy of single agent MT-3724, an ibrutinib-resistant PDX model expressing high levels of CD5 and CD20 antigens (Fig. 1h) was treated with MT-3724, resulting in significantly reduced tumor volume and increased overall survival compared with the control (Fig. 1i, j). These in vivo results indicate that MT-3724 has the potential to overcome therapeutic resistance, demonstrating the strong potential for the clinical application of this ETB in MCL treatment.

To investigate the the anti-MCL effects of MT-3724, Jeko-1, Jeko-R and Z-138 cells treated with 1000 ng/mL MT-3724 for 24 h were subjected to reverse phase protein array (RPPA), and Ingenuity Pathway Analysis (QIAGEN Bioinformatics) was performed. Overall, 63 pathways comprising 307 proteins were analyzed (Supplementary Fig. S3A and Supplementary Table S2). Jeko-1 shared three downregulated pathways with Jeko-R (PI3K/AKT signaling, mitotic roles of Polo-Like Kinase and angiopoietin signaling) and one downregulated pathway (mitotic roles of Polo-Like Kinase) with Z-138, and the three cell lines shared one downregulated pathway (mitotic roles of Polo-Like Kinase) between the negative controls and the MT-3724-treated cells lines (upper panel Supplementary Fig. S3B). Jeko-1 shared six upregulated pathways with Jeko-R and Z-138, but only one upregulated pathway (sumoylation pathway) was shared among the three cell lines (bottom panel Supplementary Fig. S3B). Jeko-1 shared more dysregulated pathways with Jeko-R than with the less sensitive cell line Z-138. We further analyzed several dysregulated pathways such as the PI3K/AKT, PTEN and apoptotic pathways, in which the components of the PI3K/AKT and apoptotic pathways were significantly different in Jeko-1 and Jeko-R cells but not in Z-138 cells (Supplementary Fig. S3C). Furthermore, several proteins (MCL-1 and cleaved caspases 3 and 7) deregulated in the RPPA analysis post-MT3724 treatment were also dysregulated in the apoptosis assays (Supplementary Fig. S2).

Ibrutinib resistance is a major therapeutic issue in MCL^21,22^; therefore, therapies to prevent and overcome resistance can have important clinical applications. We assessed the synergistic activity of ibrutinib and MT-3724 in two ibrutinib-resistant cell lines. The combination achieved strong synergy in cell viability assays, with the Ki values < 1 (Fig. 2a, b and Supplementary Fig. S4E), and in the induction of apoptosis (Fig. 2c, d and Supplementary Fig. S5A-B). We also confirmed the synergistic activity between ibrutinib and MT-3724 in additional ibrutinib-resistant cell lines (Supplementary Fig. S4A-B). Moreover, MT-3724 reduces the expression of the anti-apoptotic BCL-2 and MCL-1 (Supplementary Fig. S2C); therefore, MT-3724 and ABT-199 may synergistically induce apoptosis, serving to overcome ibrutinib resistance. We determined the synergistic activity of the combination in two ibrutinib-resistant cell lines, Granta519 (ABT-199-sensitive) and Jeko-R (ABT-199-resistant). Synergistic effects on growth inhibition (Fig. 2e, f) and apoptosis were observed (Fig. 2g, h and Supplementary Fig. S5C-D). MT-3724 and ABT-199 also had synergistic effects on two additional ibrutinib-resistant MCL cell lines, Z-138 and Maver-1 (Supplementary Fig. S4C-D). These data suggest that MT-3724 sensitizes MCL cells to ibrutinib treatment and synergistically induces cytotoxicity with ibrutinib or ABT-199.

**Fig. 2:**
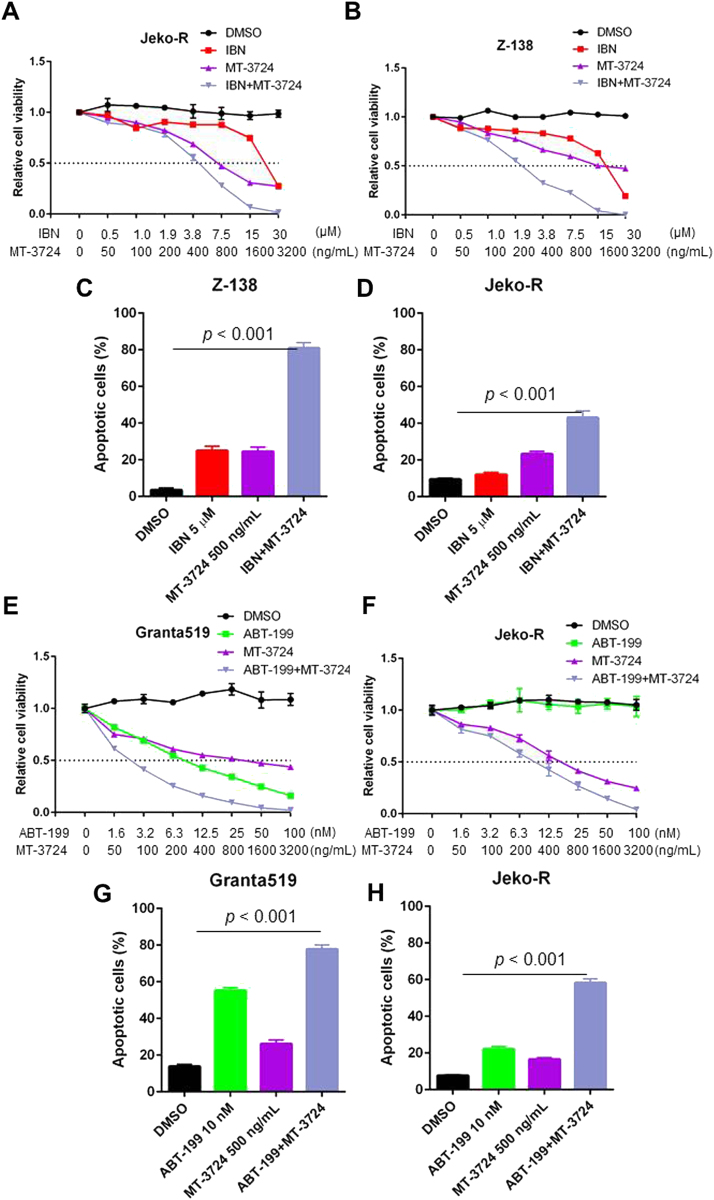
**MT-3724 in combination with ibrutinib or ABT-199 synergistically induces growth inhibition and apoptosis in therapeutic-resistant MCL cells**. **a, b** Cell viability of Jeko-R and Z-138 cells treated with single agents (MT-3724 and ibrutinib) or the combination. **c, d** Apoptosis induction of Z-138 and Jeko-R cells treated for 24 h with single agents (MT-3724 and ibrutinib) or the combination. IBN: 5 μM; MT-3724: 500 ng/mL. **e, f** Dose-dependent cell viability analysis of Granta519 and Jeko-R cells treated with single agents (MT-3724 and ABT-199) or the combination. **g, h** Apoptosis detection of Granta519 and Jeko-R cells treated for 24 h with single agents (MT-3724 and ABT-199) or the drug combination. ABT-199: 10 nM; MT-3724: 500 ng/mL. Each cell viability and apoptosis experiment was set up in triplicate and conducted two independent times

MT-3724, an engineered CD20 toxin antibody, combines targeted therapy with immunotherapy to increase efficacy. In our study, MT-3724 inhibited MCL cell growth in vitro and in vivo. All tested MCL cell lines expressed high CD20, with 6 out of 8 cell lines displaying sensitivity to MT-3724. The potential mechanisms of resistance in cells highly expressing CD20 may include mutations in CD20, structural changes affecting the binding region or alterations in the cell membrane. Post-MT-3724 treatment, the PI3K/AKT and apoptosis pathways were dysregulated in Jeko-1 and Jeko-R cells but not in the MT-3724-insensitive Z-138 cells. Several pathways were found dysregulated only in Z-138 cells, such as ILK signaling, PAK signaling, BRCA1-mediated DNA damage response and regulation of actin-based motility by Rho, suggesting that these pathways may be associated with MT-3724 resistance. Ibrutinib and venetoclax are current treatment options for MCL^13,23^; therefore, we combined MT-3724 with ibrutinib or venetoclax, which showed strong synergistic activity in both ibrutinib-resistant and venetoclax-resistant MCL cell lines. MT-3724 significantly reduced tumor burden and prolonged survival in a refractory MCL PDX mouse model, indicating that MT-3724 can reduce tumor burden in a model highly resistant to other therapies. MT-3724 has entered a Phase I/Ib study in NHL and has displayed promising single agent results^10^. Our preclinical examination of MT-3724 alone and in combination with ibrutinib or venetoclax highlights its translational relevance for MCL treatment.

## Electronic supplementary material

Supplementary Materials and Methods(DOCX 21 kb)

Supplementary Tables(DOCX 22 kb)

Supplementary Figure 1(JPG 64 kb)

Supplementary Figure 2(JPG 46 kb)

Supplementary Figure 3(JPG 110 kb)

Supplementary Figure 4(JPG 72 kb)

Supplementary Figure 5(JPG 78 kb)

Supplementary Figure Legends(DOCX 15 kb)
