# Supplementary material for: The CD20-specific engineered toxin antibody MT-3724 exhibits lethal effects against mantle cell lymphoma
Source: Blood Cancer J. 2018 Mar 20;8(3):33. doi: 10.1038/s41408-018-0066-7 (PMC5861115; doi:10.1038/s41408-018-0066-7)
Supplement: Supplementary file 1 — Supplementary Materials and Methods(DOCX 21 kb) [file 41408_2018_66_MOESM1_ESM.docx]

**Supplementary Information**

**Material and methods**

**Cell culture and reagents**

The MCL cell lines Mino, Rec-1, Maver-1 and Z-138 were purchased from ATCC (Manassas, VA, USA). Jeko-1 and Jeko-R cell lines were provided by Dr. Lan Pham at MD Anderson Cancer Center, Houston, TX. SP-49 cells were kindly provided by Dr. Jianguo Tao at Moffitt Cancer Center, Tampa, Florida. Granta-519 cells were kindly provided by Dr. Felipe Samaniego at MD Anderson Cancer Center, Houston, TX. All cell lines were cultured in RPMI 1640 medium with 10% FBS and 1% penicillin/streptomycin. MT-3724 was kindly provided by Molecular Templates in Georgetown, TX under a Materials Transfer Agreement (MTA).

**CD20 and CD77 detection by flow cytometry**

MCL cell lines (1 × 10^6^ cells) were harvested, washed twice with PBS, and re-suspended with 200 μL staining buffer (PBS + 0.1% BSA). Next, 2 μL FITC mouse anti-human CD20 (BD Biosciences, San Jose, CA, USA) was added to the cells, and the cell mixture was incubated on ice for 30 min. After incubation, the cells were washed twice with PBS and re-suspended with 200 μL staining buffer. CD20 expression was detected with the NovoCyte flow cytometer system, and the results were analyzed by FlowJo_V10.

**CellTiter-Glo® luminescent cell viability assay**

MCL cells were collected, and 10,000 cells per cell line were plated in a 96-well plate. For dose-dependent assays, 72-hour cell viability assays were conducted according to the manufacturer’s instructions (Promega, Madison, WI, USA) with 8 different doses of MT-3724 (0 to 3,200 ng/mL) and ibrutinib (0 to 30 μM). For time-dependent assays, 24-, 48- and 72-hour cell viability assays were conducted with 300 ng/mL MT-3724. Luminescence was detected with a Synergy HTX plate reader (BioTek, Winooski, VT, USA). For drug combination assays, 72-hour cell viability assays were conducted with MT-3724 (0 to 3,200 ng/mL) in combination with ibrutinib (0 to 30 μM) or ABT-199 (0 to 100 nM). Each concentration was tested in triplicate, and each experiment was performed 2-3 independent times.

**Annexin V apoptosis assay and Caspase-Glo® 3/7 luminescent assay**

Apoptosis was examined using Annexin V/PI flow cytometry and/or caspase 3/7 assays. MCL cells were treated with single agents or drug combinations for 24 hours. For the Annexin V apoptosis assay, the MCL cells were collected, washed twice with PBS and resuspended in 200 μL 1 × binding buffer. Next, FITC-conjugated Annexin V and propidium iodide (PI) were added according to the manufacturer’s instructions (BD Biosciences PharMingen). Apoptosis was detected with the NovoCyte flow cytometer system, and the results were analyzed with FlowJo_V10. Early apoptosis (annexin V^+^ PI^-^) and late apoptosis (annexin V^+^ PI^+^) were summed as total apoptosis. For the caspase 3/7 assay, treated MCL cells were resuspended in 100 μL medium, and 100 μL Caspase-Glo® 3/7 reagent was added to the suspension. Next, the mixture was incubated at room temperature for 2 hours according to the manufacturer’s instructions (Promega). Luminescence was detected with the Synergy HTX plate reader (BioTek). Each treatment was set up in triplicate, and each experiment was performed 2-3 times independently.

**Flow cytometric cell cycle analysis with propidium iodide (PI)**

Jeko-1 and Jeko-R (1 × 10^5^ cells/mL) cells were treated with 500 ng/mL MT-3724 for 24 hours. After treatment, the cells were collected, washed twice with PBS, and fixed in cold 70% ethanol for 30 min at 4°C. After fixation, the cells were washed twice with PBS and re-suspended with PBS containing 20 μg/mL RNase (Thermo Fisher Scientific, Waltham, MA, USA) and 50 ug/ mL PI (Invitrogen, Carlsbad, CA, USA). Cell cycle stages were detected with the NovoCyte flow cytometer system, and the results were analyzed by FlowJo_V10.

**Reverse phase protein array (RPPA) and KEGG pathway analysis**

RPPA experiments were conducted by the MD Anderson Cancer Center RPPA Core Facility. Briefly, 3 MCL cell lines (Jeko-1, Jeko-R and Z-138) were treated with 1,000 ng/mL MT-3724 for 24 hours, harvested and washed twice with PBS. The cell pellets were submitted to the RPPA Core Facility and subjected to RPPA analysis as previously described (20). In total, 307 antibodies and 4 secondary antibody negative controls were analyzed. IPA core analysis was performed for all 3 cells treated and untreated followed by comparison analysis. The canonical pathways with difference in activation z-score > 0.5 between untreated and treated in all 3 cells were selected for heatmap generation. The heatmap was generated in Cluster 3.0, and the resulting heatmap was visualized in Treeview and presented as a high resolution Bmp format. Each treatment was conducted in triplicate.

**Western blotting**

The MCL cell lines were with treated single agents or drug combinations for 24 hours. After treatment, the cell lines were collected and washed twice with PBS. Protein isolation was completed by RIPA lysis buffer (Thermo Fisher Scientific) with protease inhibitor cocktail (Thermo Fisher Scientific) according to the manufacturer’s instructions. Equal amounts of total protein were loaded onto 4-15% Mini-protean precast protein gels (Bio-Rad). After transferring, the membranes were blocked with 5% nonfat dry milk in 1 × TBST and then blotted with primary antibodies. Next, the membranes were blocked with secondary antibodies and developed films with regular ECL or ECL Prime (GE Healthcare Life Science). All primary antibodies (BCL-2, MCL-1, cleaved PARP, cleaved Caspase 3 and GAPDH) and secondary antibodies were purchased from Cell Signaling Technology.

**Patient-derived xenograft (PDX) model and *in vivo* drug testing**

The MCL PDX model was created by NOD SCID IL2Rγ null (NSG) mice (Jackson Laboratory) according to our previous procedures ^21, 22^. All experimental procedures and protocols were approved by The University of Texas MD Anderson Institute Animal Care Committee. In brief, fresh human fetal bones were subcutaneously implanted into NSG mice (NSG-hu). Next, 5 × 10^6^ freshly isolated cells from an MCL patient were injected into NSG-hu mice. Mouse serum was collected, and circulating human β_2_-microglobulin (β_2_M) levels were tested to monitor tumor burden (Abnova Corporation, Taipei, Taiwan). Mice were sacrificed and tumors were isolated and cut into 3mm^3^ pieces that were subcutaneously implanted into NSG mice for next generation growth. Immunophenotyping was conducted to confirm the MCL diagnosis (CD5 and CD20 expression) by flow cytometry (NovoCyte). For MT-3724 *in vivo* agent efficacy testing, PDX mice were treated *via* intraperitoneal (IP) injection with 1.2 mg/kg/dose MT-3724 (n=5) or vehicle control (PBS; n=5) for 5 days/week every other week for 4 weeks. Tumor volume by caliper measurement and survival was determined weekly.

**Statistical analysis**

The IC_50_ values were calculated by inhibition-dose-dependent analysis with GraphPad Prism 6 for each cell line. Unpaired t-test was used to compare the IC_50_ values of MT-3724 between ibrutinib-sensitive and -resistant cell lines. Statistically significant differences among treatments were determined with 2-tailed Student’s t-test. Correlation between the IC_50_ values and CD20 mean fluorescence intensity (MFI) of MT-3724 was conducted with the Analysis Toolpak add-in in Excel. A correlation coefficient near -1 indicates strong negative correlation, near 0 indicates no correlation and +1 indicates strong positive correlation. Synergy was determined by Ki = (C_A_/IC_50, A_) + (C_B_/_IC50, B_). C_A_ or C_B_ is the concentration to achieve 50% cell kill in combination while IC_50, A_ or IC_50, B_ is the concentration of IC_50_ for single agents. When Ki =1, A and B are additive; Ki < 1, A and B are synergistic; Ki > 1, A and B are antagonistic. Tumor volume was calculated with the following formula: tumor volume (mm^3^) = length (mm) × width^2^ (mm^2^)/2. Survival curves of individual groups of mice were evaluated using Kaplan-Meier analysis from the first day of treatment until sacrifice.
