# Supplementary material for: The CD20-specific engineered toxin antibody MT-3724 exhibits lethal effects against mantle cell lymphoma
Source: Blood Cancer J. 2018 Mar 20;8(3):33. doi: 10.1038/s41408-018-0066-7 (PMC5861115; doi:10.1038/s41408-018-0066-7)
Supplement: Supplementary file 2 — Supplementary Tables(DOCX 22 kb) [file 41408_2018_66_MOESM2_ESM.docx]

**Supplementary Table 1.** **IC_50_ and CD20 MFI of MT-3724 in MCL cell lines.**

| **Cell lines** | **Jeko-1** | **Mino** | **Rec-1** | **SP-49** | **Z-138** | **Maver-1** | **Jeko-R** | **Granta519** |
| --- | --- | --- | --- | --- | --- | --- | --- | --- |
| IC_50_ (ng/mL) | 139 | 272 | 426 | 78 | 1383 | 125 | 266 | 1231 |
| CD20 MFI | 9450 | 11253 | 9013 | 12365 | 5936 | 6825 | 14173 | 17639 |

**Supplementary Table 2.** **Dysregulated canonical pathways in three MCL cell lines treated with 1000 ng/ml MT-3724 for 24 hours.**

| Canonical Pathway | Jeko_  DMSO | Jeko_  MT3724 | Jeko-R_  DMSO | Jeko–R_  MT3724 | Z138_  DMSO | Z138_  MT3724 |
| --- | --- | --- | --- | --- | --- | --- |
| Angiopoietin Signaling | 0.000 | -2.121 | 1.414 | -0.707 | 0.707 | -0.707 |
| Estrogen-mediated S-phase Entry | 0.905 | 0.302 | 0.302 | -0.905 | 0.302 | -0.302 |
| ERK/MAPK Signaling | -0.557 | -1.671 | 0.557 | -0.928 | 1.300 | 0.557 |
| Cyclins and Cell Cycle Regulation | 1.000 | 0.000 | 0.500 | -1.000 | 0.000 | -0.500 |
| Aryl Hydrocarbon Receptor Signaling | 1.460 | 1.043 | 0.209 | -1.043 | -0.626 | -1.043 |
| Regulation of Actin-based Motility by Rho | 0.447 | -0.447 | -0.447 | -1.342 | 1.342 | -0.447 |
| PI3K/AKT Signaling | 1.347 | -2.117 | 0.962 | -1.347 | 0.577 | -0.577 |
| Mitotic Roles of Polo-Like Kinase | 2.236 | -0.447 | 1.342 | -2.236 | 2.236 | -0.447 |
| Colorectal Cancer Metastasis Signaling | 0.192 | -0.577 | 0.577 | -0.192 | -0.577 | -0.962 |
| ERK5 Signaling | 1.732 | 0.000 | 1.155 | 0.000 | 0.000 | -1.732 |
| PPARα/RXRα Activation | 1.213 | -0.243 | 1.213 | 0.243 | 0.243 | -0.243 |
| Sumoylation Pathway | -1.000 | 1.000 | -0.333 | 2.333 | -2.333 | -0.333 |
| Chemokine Signaling | -2.333 | -0.333 | -0.333 | 1.667 | -1.000 | -0.333 |
| PTEN Signaling | -0.655 | 1.528 | 0.218 | 1.528 | -1.528 | 0.655 |
| Activation of IRF | 0.447 | 0.447 | -0.447 | 1.342 | -1.342 | -0.447 |
| Inhibition of Angiogenesis by TSP1 | -1.134 | -0.378 | -1.134 | 1.134 | -1.134 | 0.378 |
| Neurotrophin/TRK Signaling | -1.069 | 0.535 | 0.000 | 1.069 | -1.604 | 0.000 |
| GDNF Family Ligand-Receptor Interactions | -1.069 | 0.535 | 0.000 | 1.069 | -1.604 | 0.000 |
| Apoptosis Signaling | -1.400 | 2.200 | -1.000 | 1.000 | -0.200 | 0.600 |
| 14-3-3-mediated Signaling | -0.192 | 0.577 | -0.192 | 0.962 | -1.347 | -0.962 |
| B Cell Receptor Signaling | -0.557 | -0.186 | 0.186 | 0.928 | -2.043 | -0.928 |
| PKCθ Signaling in T Lymphocytes | -0.302 | 0.905 | -0.302 | 0.905 | -1.508 | -0.302 |
| ATM Signaling | -0.894 | 0.447 | -1.789 | 0.894 | -1.342 | 0.894 |
| Neuroinflammation Signaling Pathway | -0.853 | 0.426 | 0.426 | 0.853 | -2.132 | -0.426 |
| Relaxin Signaling | -0.832 | 0.277 | -0.277 | 0.832 | -1.387 | -0.832 |
| CD40 Signaling | -1.387 | -0.277 | -0.277 | 0.832 | -0.832 | -0.277 |
| Role of Pattern Recognition Receptors | -0.277 | 0.832 | 0.277 | 0.832 | -1.387 | -0.277 |
| Role of NFAT in Regulation of the Immune Response | -0.277 | 0.277 | 0.277 | 0.832 | -1.387 | -0.832 |
| Nitric Oxide Signaling in the Cardiovascular System | 0.277 | 0.832 | -0.277 | 0.832 | -0.832 | -0.277 |
| Cytotoxic T Lymphocyte-mediated Apoptosis | -0.816 | 1.633 | 0.000 | 0.816 | -0.816 | 0.000 |
| Tumoricidal Function of Hepatic Natural Killer Cells | -0.816 | 0.816 | -0.816 | 0.816 | 0.000 | 0.816 |
| ILK Signaling | -0.784 | -0.784 | 0.392 | 0.784 | -1.569 | 0.392 |
| IL-17A Signaling in Airway Cells | -0.775 | -0.775 | -0.258 | 0.775 | -0.775 | -0.258 |
| P2Y Purigenic Receptor Signaling Pathway | -0.728 | 0.728 | 0.243 | 0.728 | -1.213 | -0.728 |
| CREB Signaling in Neurons | -0.243 | 0.243 | 0.243 | 0.728 | -1.213 | -0.243 |
| Gα12/13 Signaling | -1.606 | -0.229 | 0.229 | 0.688 | -1.147 | -0.688 |
| CXCR4 Signaling | -1.147 | -0.229 | -0.229 | 0.688 | -0.688 | 0.229 |
| NRF2-mediated Oxidative Stress Response | -1.265 | 0.000 | -1.265 | 0.632 | 0.000 | 0.632 |
| iCOS-iCOSL Signaling in T Helper Cells | 0.577 | 1.155 | 0.000 | 0.577 | -1.732 | -0.577 |
| UVC-Induced MAPK Signaling | -1.732 | 0.000 | -1.155 | 0.577 | 0.000 | 0.577 |
| FGF Signaling | -1.069 | 0.000 | -0.535 | 0.535 | -0.535 | 0.535 |
| LPS-stimulated MAPK Signaling | -1.414 | 0.943 | -0.943 | 0.471 | -0.943 | 0.943 |
| GNRH Signaling | -0.943 | 0.471 | -1.414 | 0.471 | 0.000 | 0.471 |
| Production of NO and ROS in Macrophages | -0.471 | -0.471 | -0.471 | 0.471 | -0.943 | 0.000 |
| Endothelin-1 Signaling | -1.342 | 0.894 | -0.447 | 0.447 | -1.342 | 0.894 |
| HMGB1 Signaling | -0.775 | -0.258 | -0.775 | 0.258 | -0.775 | -0.258 |
| UVB-Induced MAPK Signaling | 0.218 | 0.655 | -1.091 | 0.218 | -1.091 | 0.655 |
| Integrin Signaling | -0.218 | -0.218 | -0.218 | 0.218 | -1.091 | -0.218 |
| IL-8 Signaling | 0.577 | 0.577 | -0.192 | 0.192 | -1.347 | -0.192 |
| Toll-like Receptor Signaling | -2.000 | 0.000 | -2.000 | 0.000 | 0.000 | 1.000 |
| Cell Cycle: G2/M DNA Damage | -0.471 | 1.414 | -0.943 | 0.000 | -0.943 | -0.943 |
| TNFR1 Signaling | -1.897 | 0.000 | -0.632 | 0.000 | 0.000 | 1.265 |
| Leukocyte Extravasation Signaling | 0.000 | 0.894 | -0.447 | 0.000 | -1.789 | -0.447 |
| Antiproliferative Role of Somatostatin Receptor 2 | -0.632 | 0.632 | -0.632 | 0.000 | -1.265 | 0.000 |
| PAK Signaling | -1.000 | 0.000 | -0.500 | 0.000 | -0.500 | 1.000 |
| UVA-Induced MAPK Signaling | 0.000 | 0.000 | -1.000 | 0.000 | -1.000 | 1.000 |
| Renin-Angiotensin Signaling | -1.460 | -0.626 | -0.626 | -0.209 | -0.209 | 0.626 |
| Signaling by Rho Family GTPases | -1.043 | -1.043 | -0.626 | -0.209 | -0.209 | 0.626 |
| IL-6 Signaling | -1.606 | -1.147 | -0.688 | -0.229 | 0.229 | 0.688 |
| PDGF Signaling | -1.606 | 0.229 | -0.688 | -0.229 | -0.229 | 0.229 |
| Rac Signaling | -1.414 | -0.471 | -0.943 | -0.471 | 0.000 | 0.943 |
| EGF Signaling | -0.943 | 0.000 | -1.414 | -0.471 | 0.000 | 1.414 |
| Role of BRCA1 in DNA Damage Response | 0.632 | 0.632 | 0.000 | -0.632 | 1.265 | -1.265 |
