# Supplementary figures and images for: The CD20-specific engineered toxin antibody MT-3724 exhibits lethal effects against mantle cell lymphoma
Source: Blood Cancer J. 2018 Mar 20;8(3):33. doi: 10.1038/s41408-018-0066-7 (PMC5861115; doi:10.1038/s41408-018-0066-7)

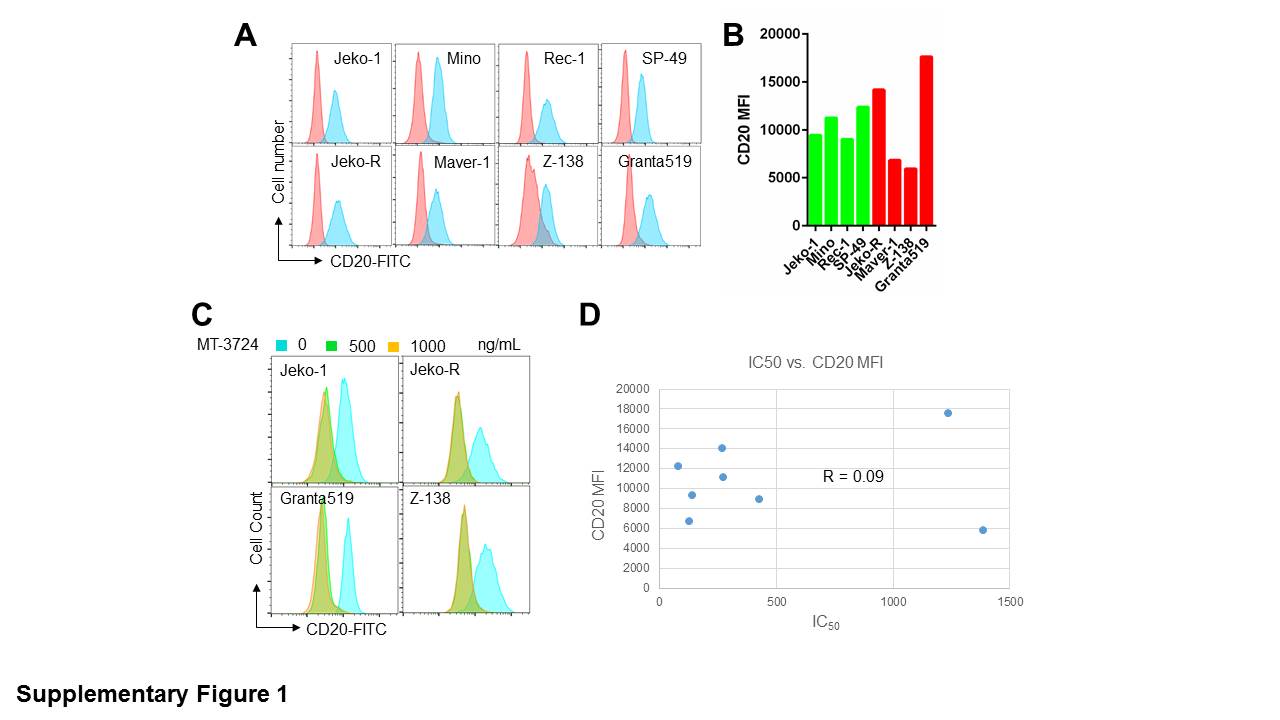

Supplement: Supplementary file 3 — Supplementary Figure 1(JPG 64 kb) [file 41408_2018_66_MOESM3_ESM.jpg]

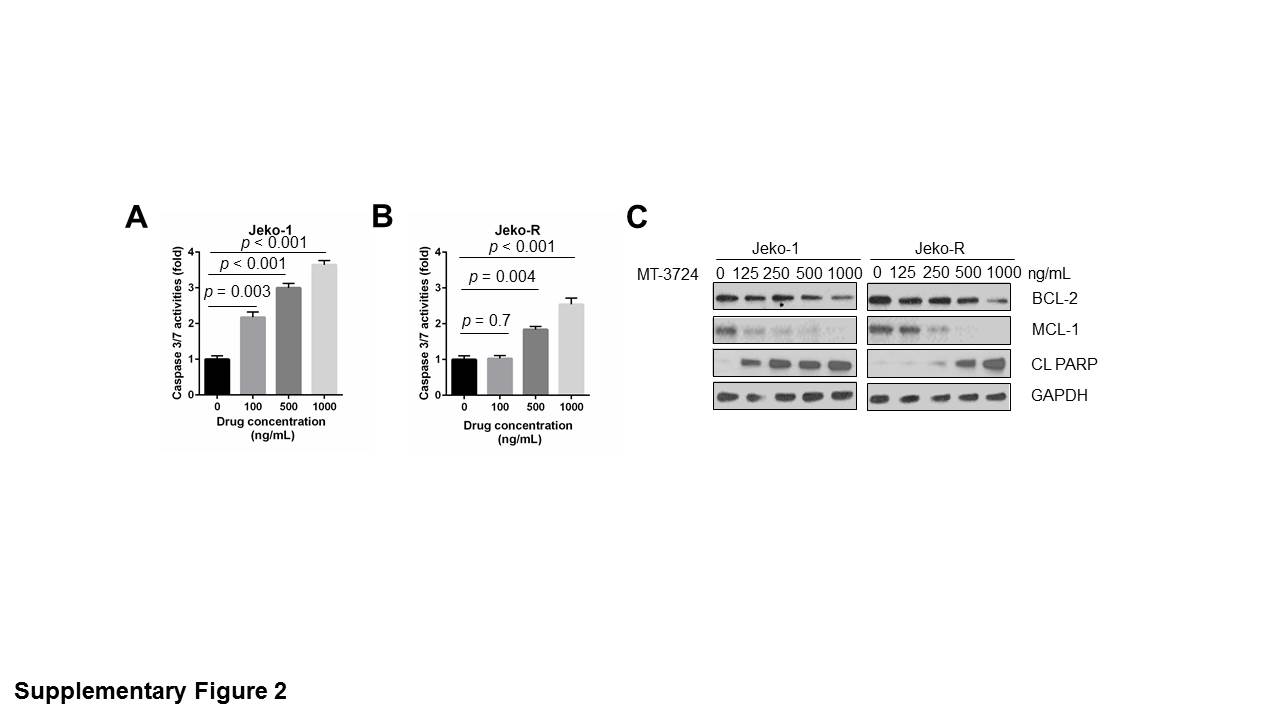

Supplement: Supplementary file 4 — Supplementary Figure 2(JPG 46 kb) [file 41408_2018_66_MOESM4_ESM.jpg]

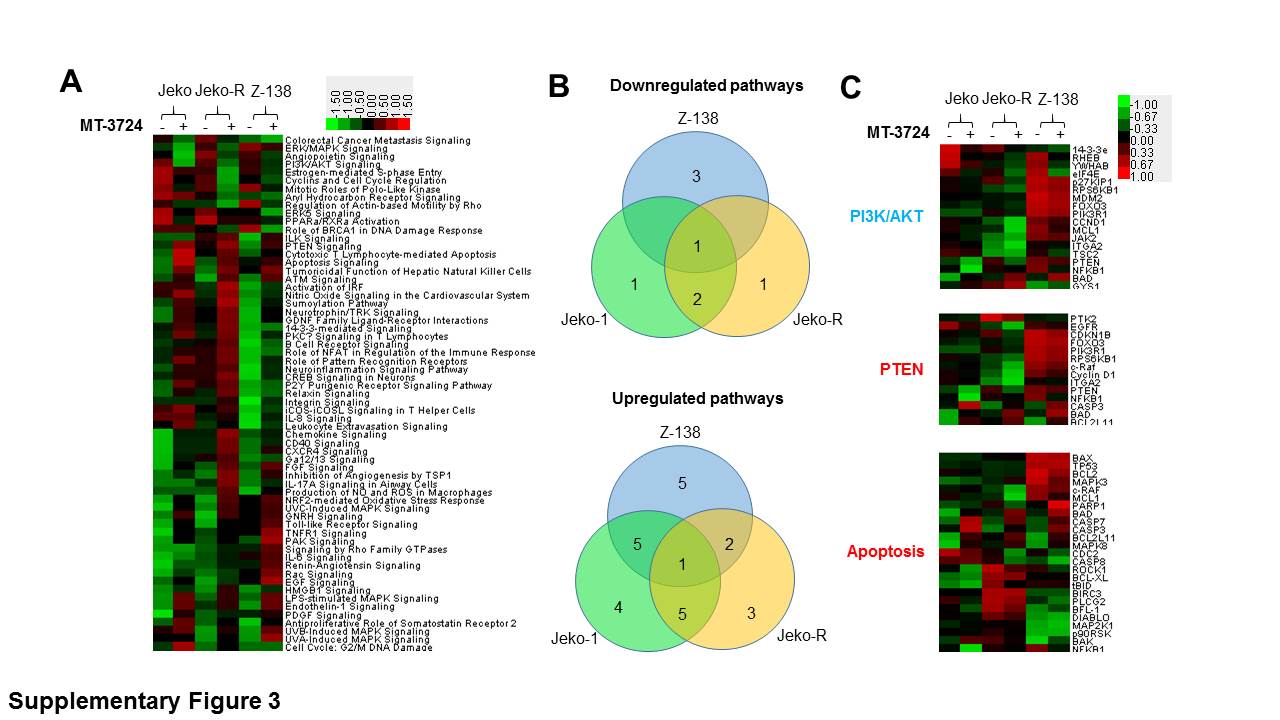

Supplement: Supplementary file 5 — Supplementary Figure 3(JPG 110 kb) [file 41408_2018_66_MOESM5_ESM.jpg]

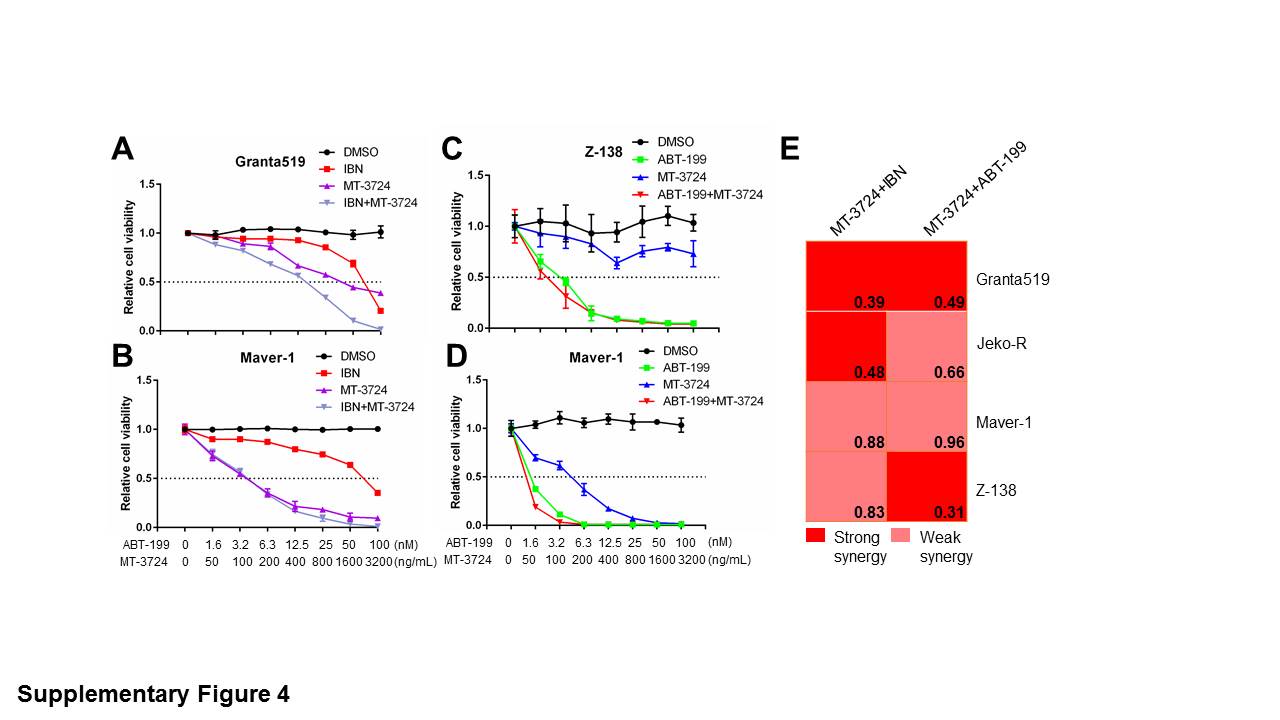

Supplement: Supplementary file 6 — Supplementary Figure 4(JPG 72 kb) [file 41408_2018_66_MOESM6_ESM.jpg]

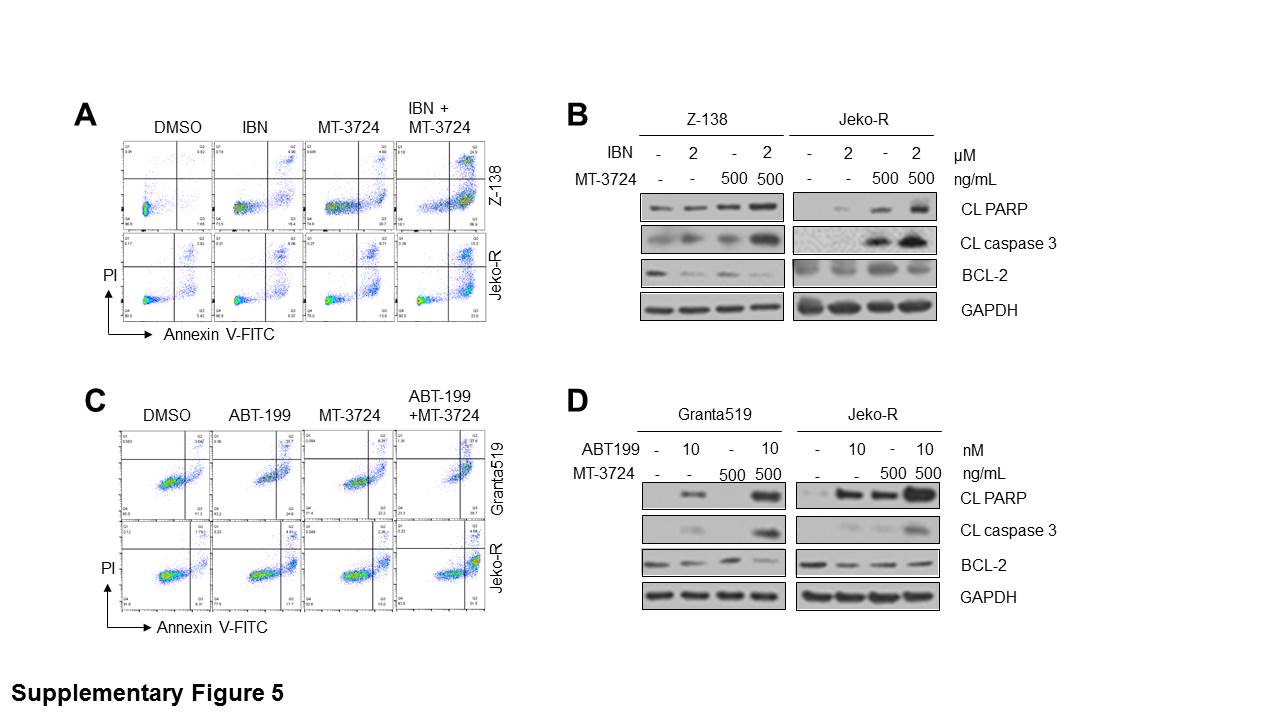

Supplement: Supplementary file 7 — Supplementary Figure 5(JPG 78 kb) [file 41408_2018_66_MOESM7_ESM.jpg]
