# Supplementary material for: The CD20-specific engineered toxin antibody MT-3724 exhibits lethal effects against mantle cell lymphoma
Source: Blood Cancer J. 2018 Mar 20;8(3):33. doi: 10.1038/s41408-018-0066-7 (PMC5861115; doi:10.1038/s41408-018-0066-7)
Supplement: Supplementary file 8 — Supplementary Figure Legends(DOCX 15 kb) [file 41408_2018_66_MOESM8_ESM.docx]

**Supplementary Figure Legends**

**Supplementary Figure 1. MT-3724 binds and blocks CD20 in MCL cells.** (**A**) CD20 expression was detected in 8 MCL cell lines by flow cytometry with a FITC-conjugated CD20 antibody (blue) and an isotype control (red). (**B**) CD20 MFI in 8 MCL cell lines determined by subtracting the isotype background. (**C**) CD20 detection in 4 MCL cell lines treated with the indicated doses of MT-3724 for 24 hours. CD20 was detected by flow cytometry. (**D**) Correlation between IC_50_ and CD20 MFI of MT-3724 calculated with the CORREL function in Excel, Microsoft. Coefficient (a value between -1 and +1) denotes the relationship between the two variables.

**Supplementary Figure 2. MT-3724 induces apoptosis in both ibrutinib-sensitive and -resistant cells.** (**A-B**) Caspase 3/7 luminescence was conducted to detect caspase 3 and 7 levels after MT-3724 treatment using an Annexin V/PI assay. (**C**) Immunoblotting of BCL-2, MCL-1 and cleaved PARP in Jeko-1 and Jeko-R cells treated with the indicated 5 doses of MT-3724 for 24 hours. GAPDH served as a loading control.

**Supplementary Figure 3.** **Mechanisms mediating MT-3724 activity in ibrutinib-sensitive and –resistant MCL cell lines.** (**A**) Jeko-1, Jeko-R and Z-138 cells were treated with 1,000 ng/mL for 24 hours, and RPPA analysis was conducted based on NormLog2_MedianCentered values as described in the Materials and Methods. IPA core analysis were performed for all 3 cells treated and untreated followed by comparison analysis. The canonical pathways with difference in activation z-score >0.5 between untreated and treated in all 3 cells were selected for heatmap generation. (**B**) Statistical analysis of dysregulated pathways shared among 3 MCL cell lines. (**C**) Heatmap of 3 dysregulated pathways among 3 MCL cell lines between pre- and post-treatment of MT-3724.

**Supplementary Figure 4.** **MT-3724 synergistically acts with ibrutinib and ABT-199 in ibrutinib-resistant cell lines.** (**A-B**) Cell viability analysis of Granta519 and Maver-1 cells treated at the indicated doses of MT-3724 and ibrutinib single agents or the combination. (**C-D**) Cell viability of Granta519 and Maver-1 cells treated with MT-3724 and ABT-199 single agents or the combination. (**E**) Synergistic heatmap generated based on Ki value. Strong synergy is denoted with red, and weak synergy is denoted with pink.

**Supplementary Figure 5. Combination of MT-3724 and ibrutinib or ABT-199 synergistically induces apoptosis.** (**A**) Z-138 and Jeko-R were treated for 24 hours with single agents (MT-3724 and ibrutinib) or the drug combination, and apoptosis was detected by Annexin V/PI staining. (**B**) Immunoblotting of cleaved PARP, caspase 3 and BCL-2 using whole cell extracts from (**A**). (**C**) Granta519 and Jeko-R were treated for 24 hours with single agents (MT-3724 and ABT-199) or drug combination, and apoptosis was detected by Annexin V/PI staining. (**D**) Immunoblotting of cleaved PARP, caspase 3 and BCL-2 using whole cell extracts from (**C**).
